# Heart Failure With Preserved Ejection Fraction‐Like Phenotype in Coronary Artery Disease and Obstructive Sleep Apnea: Insights From the RICCADSA Cohort

**DOI:** 10.1002/clc.70402

**Published:** 2026-06-29

**Authors:** Erik Thunström, Helena Glantz, Aylin Pihtili, Yüksel Peker

**Affiliations:** ^1^ Department of Molecular and Clinical Medicine, Institute of Medicine, Sahlgrenska Academy University of Gothenburg Gothenburg Sweden; ^2^ Department of Medicine, Geriatrics and Emergency Medicine, Region Västra Götaland Sahlgrenska University Hospital/Östra Gothenburg Sweden; ^3^ Department of Internal Medicine Skaraborg Hospital Lidköping Sweden; ^4^ Department of Pulmonary Medicine, School of Medicine Istanbul University Istanbul Türkiye; ^5^ Department of Pulmonary Medicine Koc University School of Medicine Istanbul Türkiye; ^6^ Division of Pulmonary, Allergy, and Critical Care Medicine University of Pittsburgh School of Medicine Pittsburgh Pennsylvania USA; ^7^ Department of Clinical Sciences, Respiratory Medicine and Allergology, Faculty of Medicine Lund University Lund Sweden

**Keywords:** aging, coronary artery disease, HFpEF, obesity, obstructive sleep apnea

## Abstract

**Background:**

Heart failure with preserved ejection fraction (HFpEF) is closely linked to aging and cardiometabolic risk factors and frequently coexists with obstructive sleep apnea (OSA). We aimed to investigate the prevalence and clinical correlates of an HFpEF‐like phenotype in a revascularized coronary artery disease (CAD cohort), focusing on OSA and its severity.

**Methods:**

A total of 435 patients with preserved left ventricular ejection fraction from the RICCADSA cohort were included. OSA was defined as apnea–hypopnea index (AHI) ≥ 15 events/h. HFpEF‐like phenotype was defined by ≥ 2 of the following: elevated filling pressures (E/e′ ≥ 15), left atrial enlargement, increased left ventricular mass index, elevated pulmonary artery systolic pressure (≥ 35 mmHg), and elevated NT‐proBNP (≥ 125 pg/mL). Multivariable logistic regression analyses included age, sex, obesity, hypertension, diabetes, and OSA status. Additional models evaluated AHI and oxygen desaturation index (ODI) as continuous variables and assessed the impact of excluding body mass index (BMI).

**Results:**

Mean age was 63.6 ± 8.6 years and BMI 28.1 ± 4.1 kg/m^2^; 69.9% met HFpEF‐like criteria. Age and obesity were independently associated with HFpEF‐like phenotype, whereas categorical OSA was not. AHI and ODI were not independently associated after adjustment; however, in models excluding BMI, both AHI (OR 1.019, 95% CI 1.005–1.033) and ODI (OR 1.030, 95% CI 1.010–1.050) were significant predictors.

**Conclusions:**

HFpEF‐like phenotype is highly prevalent in CAD and primarily associated with aging and adiposity. The relationship between OSA severity and cardiac remodeling appears dependent on obesity, underscoring the interplay between cardiometabolic and sleep‐related factors.

**Pre‐registered Clinical Trial Number:**

The RICCADSA trial is registered at ClinicalTrials.gov (NCT00519597) and in the Swedish national research registry (FoU i Sverige—Research and Development in Sweden; registration no. VGSKAS‐4731; April 29, 2005).

## Graphical abstract

HFpEF‐like cardiac remodeling was common in patients with coronary artery disease and preserved ejection fraction. Aging and obesity emerged as the principal correlates, while the contribution of sleep‐disordered breathing severity appeared modest and closely intertwined with adiposity.

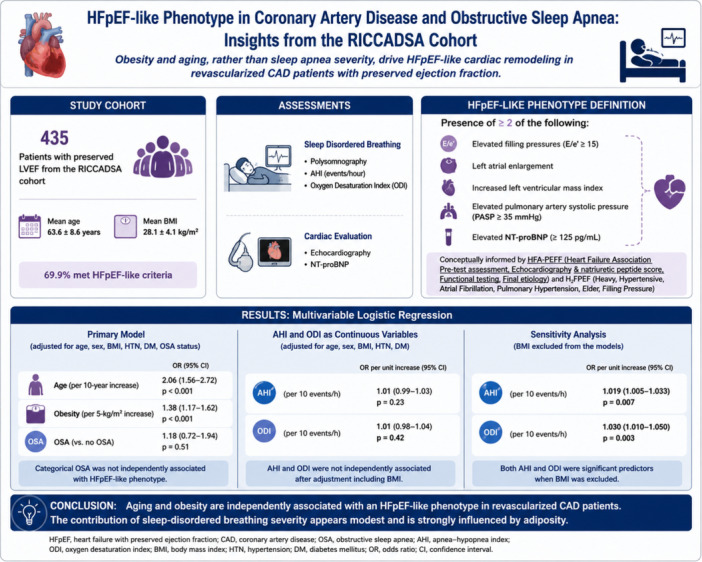

## 1 Introduction

Heart failure with preserved ejection fraction (HFpEF) has emerged as a major public health challenge and now represents approximately half of all heart failure cases, particularly among older adults [1, 2]. The prevalence of HFpEF increases markedly with aging and is expected to rise further with demographic changes and the growing burden of cardiometabolic disease [3, 4]. Epidemiological studies indicate that HFpEF is the predominant form of heart failure in the elderly population, and its incidence doubles with each decade after the age of 65 [5, 6, 7].

HFpEF is increasingly recognized as a heterogeneous syndrome driven by multiple interacting pathophysiological mechanisms, including systemic inflammation, endothelial dysfunction, myocardial fibrosis, and impaired ventricular relaxation [8, 9, 10, 11]. Among these, obesity and metabolic comorbidities have emerged as central contributors [12]. A substantial proportion of patients with HFpEF are overweight or obese, and epidemiological data demonstrate a strong independent association between excess adiposity and the development of HFpEF [12, 13, 14]. The cardiometabolic phenotype—characterized by obesity, hypertension, diabetes, and dyslipidemia—appears to play a pivotal role in the clinical expression of the syndrome [8, 9, 12, 13, 14, 15].

Obstructive sleep apnea (OSA) frequently coexists with HFpEF and shares common risk factors such as aging, obesity, and metabolic dysfunction. Recurrent nocturnal hypoxia, sympathetic activation, and intrathoracic pressure changes associated with OSA may contribute to diastolic dysfunction and adverse cardiac remodeling [16, 17, 18, 19]. Observational studies have reported associations between OSA severity and markers of diastolic dysfunction and HFpEF probability scores; however, these relationships appear to be strongly influenced by underlying obesity and cardiometabolic burden [20].

Despite increasing recognition of HFpEF as a cardiometabolic syndrome, the determinants of HFpEF‐like characteristics in populations with varying OSA status remain incompletely understood. In particular, it is unclear whether OSA independently contributes to HFpEF‐like phenotypes beyond shared risk factors such as age and obesity.

Therefore, the present study aimed to investigate the prevalence and clinical correlates of an HFpEF‐like phenotype in the RICCADSA cohort, with a specific focus on the relative contributions of age, obesity, cardiometabolic comorbidities, and OSA status.

## 2 Materials and Methods

### 2.1 Study Design and Population

The present study represents a secondary observational analysis derived from the RICCADSA cohort, an investigator‐initiated randomized controlled trial conducted at a single center in Sweden. The overall design and primary outcomes of the RICCADSA study have been described previously [21].

**Figure 1:**
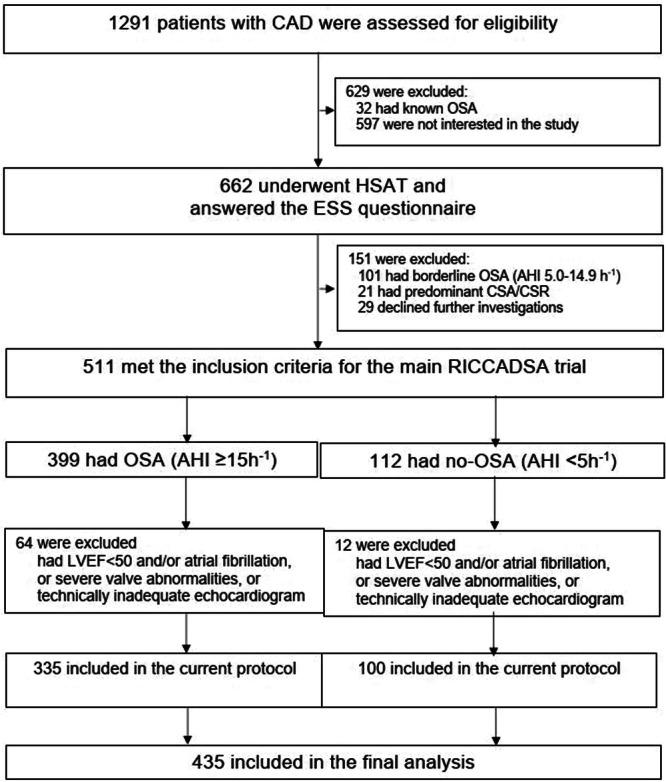
Flowchart showing participant screening, eligibility assessment, and inclusion in the final analysis.

As illustrated in Figure 1, patients with established coronary artery disease (CAD) who had undergone prior revascularization were screened for sleep‐disordered breathing. Individuals with preserved left ventricular ejection fraction (LVEF) (≥ 50%) and available echocardiographic, sleep, and biomarker data were eligible for inclusion in the present analysis. Participants with a history of atrial fibrillation were excluded.

The study protocol was approved by the Ethics Committee of the Medical Faculty at the University of Gothenburg (approval no. 207‐05; September 13, 2005), and all participants provided written informed consent. The RICCADSA trial is registered at ClinicalTrials.gov (NCT00519597) and in the Swedish national research registry (FoU i Sverige—Research and Development in Sweden; registration no. VGSKAS‐4731; April 29, 2005).

### 2.2 Sleep Assessment

Sleep‐disordered breathing was evaluated using unattended home sleep apnea testing (HSAT) with a portable monitoring system (Embletta, Embla, Broomfield, CO, USA). Recordings included measurements of airflow, thoracoabdominal respiratory effort, oxygen saturation, body position, and heart rate.

Apneas and hypopneas were scored according to established criteria, and the apnea–hypopnea index (AHI) and oxygen desaturation index (ODI) were calculated as the number of events per hour of recording [22]. Daytime sleepiness was assessed using the Epworth Sleepiness Scale (ESS) [23].

### 2.3 Clinical Characteristics and Comorbidities

Demographic variables, anthropometric measurements, cardiovascular risk factors, and comorbid conditions were obtained from medical records and standardized study assessments. Body mass index (BMI) was calculated as weight divided by height squared (kg/m^2^), and obesity was defined as BMI ≥ 30 kg/m^2^.

### 2.4 Echocardiographic Assessment

Transthoracic echocardiography was performed using a commercially available ultrasound system by experienced sonographers. Standard parasternal and apical views were acquired at rest and analyzed offline using dedicated software.

Measurements included left ventricular dimensions, wall thickness, and left ventricular mass. Left ventricular mass index (LVMI) was calculated and indexed to height.^2.7^ Left atrial size, LVEF, transmitral flow velocities (E and A), and tissue Doppler–derived early diastolic velocity (e′) were recorded. The E/e′ ratio was calculated as an estimate of left ventricular filling pressure [24, 25].

Pulmonary artery systolic pressure was estimated from tricuspid regurgitation velocity when available [26].

### 2.5 Biomarker Assessment

Venous blood samples were obtained in the morning following sleep recordings. Plasma concentrations of N‐terminal pro–brain natriuretic peptide (NT‐proBNP) were measured using standardized immunoassays. For statistical analyses, NT‐proBNP values were log‐transformed.

### 2.6 Definition of HFpEF‐Like Phenotype

HFpEF‐like status was defined using a composite classification based on echocardiographic and biomarker criteria aligned with contemporary HFpEF diagnostic frameworks. Participants were classified as having an HFpEF‐like phenotype if at least two of the following criteria were present [3]: evidence of elevated filling pressures (septal E/e′ ≥ 15), left atrial enlargement (left atrial diameter ≥ 40 mm in men or ≥ 39 mm in women), increased LVMI (≥ 49 g/m^2.7^ in men or ≥ 45 g/m^2.7^ in women), elevated pulmonary artery systolic pressure (≥ 35 mmHg), and increased natriuretic peptide levels (NT‐proBNP ≥ 125 pg/mL). The term “HFpEF‐like phenotype” was used to describe structural and biomarker abnormalities consistent with HFpEF‐related cardiac remodeling rather than clinically overt HFpEF, as symptoms and functional limitation were not assessed in this analysis.

### 2.7 Statistical Analysis

The present analysis represents a secondary cross‐sectional analysis of the RICCADSA cohort, and the sample size was determined by the number of participants with complete echocardiographic, biomarker, and sleep study data available for analysis. Continuous variables are presented as mean ± standard deviation, and categorical variables as counts and percentages. Between‐group comparisons were performed using independent samples *t*‐tests for continuous variables and chi‐square tests for categorical variables.

To identify independent correlates of the HFpEF‐like phenotype, multivariable logistic regression analyses were performed. The primary model included age category, sex, BMI ≥ 30 kg/m^2^, hypertension, diabetes, and OSA status. Potential interaction effects between OSA and key cardiometabolic variables were explored in additional analyses. Model calibration was assessed using the Hosmer–Lemeshow goodness‐of‐fit test, and model discrimination was evaluated using the area under the receiver operating characteristic curve (AUC). Potential multicollinearity among predictor variables was evaluated using collinearity diagnostics and variance inflation factors.

Results are reported as odds ratios (OR) with 95% confidence intervals (CI). A two‐sided *p* < 0.05 was considered statistically significant.

All statistical analyses were conducted using IBM SPSS Statistics (version 28.0; IBM Corp., Armonk, NY, USA).

## 3 Results

As shown in Figure 1, a total of 435 participants from the RICCADSA cohort were included. The mean age was 63.6 ± 8.6 years, and the mean BMI was 28.1 ± 4.1 kg/m^2^. Overall, 304 participants (69.9%) met criteria for an HFpEF‐like phenotype.

As presented in Table 1, participants with HFpEF‐like phenotype were older and had higher BMI compared with those without HFpEF‐like features. Waist–hip ratio was also higher in the HFpEF‐like group, indicating greater central adiposity. Comorbid conditions were broadly similar between groups, whereas sleep‐related parameters were modestly higher in the HFpEF‐like group. Analyses of nocturnal hypoxic burden showed that mean nocturnal oxygen saturation and T90 were similar between groups, while nadir oxygen saturation was modestly lower in participants with HFpEF‐like phenotype.

**Table 1:** Baseline characteristics according to HFpEF‐like phenotype.

Variable	HFpEF‐like (*n* = 304)	Non‐HFpEF (*n* = 131)	*p* value
Demographics			
Age, years	64.6 ± 8.3	61.2 ± 8.8	< 0.001
Female sex, *n* (%)	52 (17.1)	26 (19.8)	0.494
Anthropometrics			
BMI, kg/m^2^	28.6 ± 4.2	27.1 ± 3.4	< 0.001
BMI ≥ 30 kg/m^2^, *n* (%)	88 (28.9)	24 (18.3)	0.020
Waist‐hip‐ratio	1.01 ± 0.5	0.9 ± 0.1	0.037
Comorbidities			
Hypertension, *n* (%)	188 (61.8)	67 (51.1)	0.038
Diabetes mellitus, *n* (%)	65 (21.4)	28 (21.4)	1.000
Acute myocardial infarction, *n* (%)	153 (50.3)	65 (49.6)	0.999
Stroke history, *n* (%)	20 (6.7)	2 (1.5)	0.026
Lung disease, *n* (%)	25 (8.2)	13 (9.9)	0.565
Sleep parameters			
OSA (yes), *n* (%)	245 (80.6)	90 (68.7)	0.007
ESS score	7.6 ± 4.0	7.7 ± 3.8	0.740
AHI events/h	25.4 ± 17.6	19.3 ± 15.2	< 0.001
ODI events/h	15.7 ± 14.9	10.5 ± 11.1	< 0.001
Mean SpO_2,_ %	93.4 ± 3.0	93.8 ± 3.7	0.243
Nadir SpO_2,_ %	81.7 ± 8.6	84.9 ± 6.6	< 0.001
SpO2 time spent < 90%, min	8.4 ± 17.8	7.0 ± 19.0	0.521
Echocardiographic parameters			
LVEF, %	59.5 ± 4.9	61.4 ± 4.5	—
Left atrial volume, mm	44.1 ± 5.0	39.2 ± 4.9	—
E/e′, ratio	11.5 ± 3.7	9.4 ± 2.3	—
LV mass index, g/m^2.7^	55.0 ± 13.5	41.0 ± 8.0	—
Pulmonary arterial pressure, mmHg	30.8 ± 5.8	27.6 ± 3.6	—
Biomarkers			
ln(NT‐proBNP)	5.52 ± 0.96	4.52 ± 0.82	—

*Note:* Data are presented as mean ± standard deviation or *n* (%). Comparisons were performed using an independent samples *t*‐test or chi‐square test, as appropriate. Echocardiographic and biomarker variables used to define the HFpEF‐like phenotype are presented descriptively and were not statistically compared between groups.

Abbreviations: AHI, apnea‐hypopnea‐index; BMI, body‐mass‐index; E, transmitral flow velocity; é, early diastolic velocity; ESS, Epworth sleepiness scale; HFpEF, heart failure with preserved ejection fraction; LV, left ventricular; LVEF, left ventricular ejection fraction; ln, natural logarithm; NT‐proBNP, N‐terminal pro–brain natriuretic peptide; ODI, oxygen desaturation index; OSA, obstructive sleep apnea; SpO_2_, oxygen saturation.

As expected by definition, participants classified as HFpEF‐like more frequently exhibited echocardiographic and biomarker abnormalities consistent with elevated filling pressures, structural cardiac remodeling, and increased natriuretic peptide levels. These parameters were integral components of the HFpEF‐like definition and are therefore presented descriptively. Distribution of individual HFpEF‐like phenotype components is illustrated in Table 2.

**Table 2:** Distribution of individual HFpEF‐like phenotype components.

Variable	HFpEF‐like (*n* = 304)	Non‐HFpEF (*n* = 131)
Increased left atrial volume, %	87.8	40.8
Increased E/e′ ratio, %	16.3	0.8
Elevated LV mass index, %	70.1	9.2
Increased pulmonary arterial pressure, %	9.9	0.8
Elevated NT‐proBNP, %	81.8	24.4

*Note:* Data are presented as percentages.

Abbreviations: E, transmitral flow velocity; é, early diastolic velocity; HFpEF, heart failure with preserved ejection fraction; LV, left ventricular; NT‐proBNP, N‐terminal pro–brain natriuretic peptide.

OSA was common in the overall cohort. In unadjusted analyses, HFpEF‐like phenotype appeared more frequent among participants with OSA compared with those without OSA; however, this association did not remain significant after adjustment for clinical covariates.

**Figure 2:**
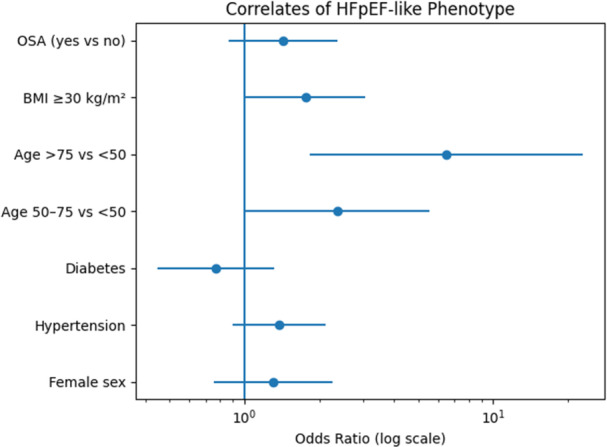
Multivariable logistic regression analysis showing clinical correlates of the HFpEF‐like phenotype. Odds ratios and 95% confidence intervals are presented on a logarithmic scale.

In multivariable logistic regression analysis, increasing age and obesity were independently associated with HFpEF‐like phenotype (Figure 2). The model showed good calibration (Hosmer–Lemeshow *χ* ^2^ = 6.27, *p* = 0.617) and acceptable discrimination (AUC = 0.74). Compared with participants aged < 50 years, those aged 50–75 years had an OR of 2.36 (95% CI 1.01–5.54; *p* = 0.049), and those aged > 75 years had an OR of 6.51 (95% CI 1.84–23.01; *p* = 0.004). Obesity was also independently associated (OR 1.76, 95% CI 1.01–3.06; *p* = 0.045). Sex, hypertension, diabetes, and OSA status were not significant predictors. Variance inflation factors were < 2 for all variables, indicating no evidence of multicollinearity.

When age was modeled as a continuous variable, increasing age remained independently associated with HFpEF‐like phenotype (OR 1.053 per year, 95% CI 1.024–1.086; *p* < 0.001). BMI also remained an independent predictor, whereas OSA‐related variables were not significantly associated.

To address potential overadjustment by obesity, an additional model excluding BMI was performed. In this model, both AHI (OR 1.019, 95% CI 1.005–1.033; *p* = 0.007) and ODI (OR 1.030, 95% CI 1.010–1.050; *p* = 0.003) were independently associated with HFpEF‐like phenotype.

In contrast, in models including BMI, neither AHI nor ODI remained independently associated (AHI: OR 1.011, 95% CI 0.996–1.026; *p* = 0.141; ODI: OR 1.017, 95% CI 0.997–1.038; *p* = 0.099).

No significant interactions were observed between sleep apnea metrics and BMI or age (all *p* > 0.05).

## 4 Discussion

In this analysis of the RICCADSA cohort, the HFpEF‐like phenotype was highly prevalent and strongly associated with increasing age and adiposity. A key finding of the present study is that the relationship between OSA and HFpEF‐like cardiac remodeling varied according to model specification. While OSA status defined by conventional thresholds was not independently associated with HFpEF‐like phenotype, the association between OSA severity and cardiac remodeling was attenuated after adjustment for cardiometabolic factors, particularly obesity.

This observation suggests that the contribution of OSA to HFpEF‐like cardiac changes is closely intertwined with adiposity. In models including BMI, neither AHI nor ODI remained independently associated, whereas in sensitivity analyses excluding BMI, both parameters emerged as significant predictors. These findings indicate that the effect of intermittent hypoxia and respiratory disturbances may be partly masked or mediated by obesity, which is strongly linked to both OSA severity and cardiac structural remodeling.

The relatively high prevalence of HFpEF‐like phenotype observed in the present cohort likely reflects the high‐risk characteristics of the study population. Previous studies using contemporary diagnostic frameworks and risk‐based approaches have reported a high prevalence of HFpEF‐related structural and functional abnormalities in cardiometabolic populations, particularly when composite criteria or probability‐based scores are applied [3, 13, 14, 27]. These approaches are designed to capture early cardiac remodeling and therefore yield higher prevalence estimates compared with clinically overt HFpEF. Our HFpEF‐like classification was conceptually informed by contemporary diagnostic frameworks such as the HFA‐PEFF (Heart Failure Association–Pre‐test assessment, Echocardiography and natriuretic peptide score, Functional testing, Final etiology) and H_2_FPEF (Heavy, Hypertensive, Atrial Fibrillation, Pulmonary Hypertension, Elder, Filling Pressure) algorithms, both of which incorporate structural cardiac abnormalities, elevated filling pressures, natriuretic peptide levels, obesity, and other cardiometabolic characteristics [3, 27]. However, unlike these diagnostic approaches, symptom burden and functional limitation were not assessed in the present study. Therefore, the phenotype identified herein should be interpreted as reflecting HFpEF‐related cardiac remodeling rather than clinically established HFpEF.

HFpEF is increasingly recognized as the dominant form of heart failure among older adults and individuals with cardiometabolic disease. Population‐based studies demonstrate that aging is one of the strongest determinants of HFpEF, with prevalence rising steeply after the sixth decade of life [5, 6, 7]. Age‐related myocardial stiffening, vascular dysfunction, and systemic inflammation are thought to contribute to impaired ventricular relaxation and elevated filling pressures, hallmarks of HFpEF physiology [7, 8, 9, 10, 11]. In the present cohort, the strong gradient observed across age categories is consistent with these mechanisms and reinforces the role of biological aging as a central driver of HFpEF‐like characteristics.

Obesity emerged as another key correlate of HFpEF‐like status. Excess adiposity has been linked to myocardial remodeling, systemic inflammation, endothelial dysfunction, and increased circulating blood volume, all of which may promote diastolic dysfunction and elevated cardiac filling pressures [12, 13, 14]. Contemporary HFpEF frameworks increasingly describe an “obese cardiometabolic phenotype,” characterized by the clustering of obesity, hypertension, insulin resistance, and low‐grade inflammation [3, 9]. Our findings are consistent with this model and further suggest that obesity may play a central role in modulating the relationship between OSA and cardiac remodeling. From a causal perspective, obesity may function as both a confounder and a mediator linking sleep‐disordered breathing to HFpEF‐related cardiac remodeling. Consequently, adjustment for BMI may attenuate associations attributable to shared biological pathways, whereas omission of BMI may overestimate the direct contribution of sleep‐disordered breathing. The differing results across models should therefore be interpreted within this context.

Although OSA is frequently associated with HFpEF and shares multiple risk factors—including age, obesity, and cardiometabolic disease—its independent contribution remains debated. Recurrent nocturnal hypoxia, sympathetic activation, and intrathoracic pressure swings have been proposed as mechanisms linking OSA to diastolic dysfunction and cardiac remodeling [16, 17, 18, 19]. Observational studies have reported associations between OSA severity and HFpEF probability scores; however, these relationships often attenuate after adjustment for obesity and comorbid conditions [20, 27]. The present findings align with these observations and suggest that the contribution of sleep‐disordered breathing to HFpEF‐like cardiac remodeling is modest and largely dependent on the underlying cardiometabolic context.

Notably, parameters reflecting nocturnal hypoxic burden, including mean oxygen saturation and T90, were not significantly different between groups, while nadir oxygen saturation showed only modest differences. These findings further support the notion that conventional OSA metrics may not fully capture the complex pathophysiological pathways linking OSA with cardiac remodeling in this population.

These findings have potential clinical implications. First, they emphasize the importance of cardiometabolic risk profiling in patients with suspected HFpEF, particularly among those with preserved systolic function but multiple metabolic comorbidities. Second, they suggest that the presence of OSA alone may not sufficiently explain HFpEF‐like cardiac alterations without concomitant aging and obesity‐related mechanisms. This reinforces the need for integrated management strategies targeting weight reduction, metabolic control, and cardiovascular risk modification in addition to OSA treatment.

The present study should be interpreted in the context of several limitations. The analysis was observational and derived from a single‐center cohort consisting predominantly of male patients with revascularized CAD and a high prevalence of OSA, which may limit the generalizability of the findings to broader cardiovascular populations. Moreover, a cross‐sectional design precludes conclusions regarding temporal or causal relationships. The HFpEF‐like classification relied on echocardiographic and biomarker criteria rather than clinical heart failure diagnosis, and longitudinal outcome data were not evaluated in this analysis. Residual confounding by unmeasured cardiometabolic factors cannot be excluded. In addition, the study population consisted of patients with established CAD, which may influence the prevalence and phenotypic expression of HFpEF‐like features. Another limitation relates to the use of HSAT. Although cardiorespiratory polygraphy allows differentiation between obstructive and central respiratory events based on airflow and thoracoabdominal movement signals, it does not provide the full physiological detail of in‐laboratory polysomnography. Consequently, subtle or intermittent central respiratory events may not be fully characterized, which could introduce some heterogeneity and potentially attenuate the observed associations between OSA and HFpEF‐like phenotype. Importantly, the phenotype examined in the present study represents structural and biomarker abnormalities consistent with HFpEF‐related cardiac remodeling rather than clinically overt HFpEF, as symptom status and functional limitation were not assessed. Moreover, the cross‐sectional design precludes conclusions regarding temporal or causal relationships between OSA, cardiometabolic factors, and HFpEF‐like cardiac remodeling.

Despite these limitations, the study provides insight into the relative contributions of aging, obesity, and sleep‐disordered breathing to HFpEF‐like characteristics in a well‐phenotyped cohort. The findings support the concept that HFpEF‐like cardiac remodeling is predominantly driven by cardiometabolic and age‐related mechanisms, with a modest contribution from sleep‐disordered breathing that appears closely linked to adiposity.

In conclusion, an HFpEF‐like phenotype was highly prevalent in this CAD cohort and was primarily associated with aging and obesity. While OSA, defined as a categorical variable, was not independently associated with HFpEF‐like phenotype, the contribution of sleep‐disordered breathing severity appears modest and strongly influenced by adiposity, highlighting the complex interplay between metabolic and respiratory factors in HFpEF‐related cardiac remodeling.

## Author Contributions

Concept, study design, drafting the work: Erik Thunström. Concept, study design, acquisition, drafting the work: Helena Glantz. Concept, study design, drafting the work: Aylin Pihtili. Concept, study design, analysis, drafting the work: Yüksel Peker. All authors interpreted the data. Yüksel Peker obtained study funding and takes full responsibility for the work, including the study design, access to data, and the decision to submit and publish the manuscript. All authors approved this manuscript in its final form.

## Ethics Statement

The study protocol was approved by the Ethics Committee of the Medical Faculty at the University of Gothenburg (approval no. 207‐05; September 13, 2005), with subsequent amendments (T744‐10; November 26, 2010, and T512‐11; June 16, 2011). Written informed consent was obtained from all participants.

## Conflicts of Interest

Y.P. received institutional grants from the ResMed Foundation for the main RICCADSA trial. The other authors declare no conflicts of interest.

## Data Availability

Data collected for the study, including de‐identified individual participant data will be made available to others within 6 months after the publication of this article, as will additional related documents (study protocol, statistical analysis plan, and informed consent form), for academic purposes (e.g., meta‐analyses), upon request to the corresponding author (yuksel.peker@lungall.gu.se), and with a signed data access agreement.
